# *Epidermal Patterning Factor 2-like* (*McEPFL2*): A Putative Candidate for the Continuous Ridge (cr) Fruit Skin Locus in Bitter Gourd (*Momordica charantia* L.)

**DOI:** 10.3390/genes13071148

**Published:** 2022-06-25

**Authors:** Jing Yang, Yiqun Weng, Huihong Li, Qiusheng Kong, Weiluan Wang, Chenghuan Yan, Liping Wang

**Affiliations:** 1 Key Laboratory of Horticultural Plant Biology, Ministry of Education, College of Horticulture and Forestry Sciences, Huazhong Agricultural University, Wuhan 430070, China; yangjing80@mail.hzau.edu.cn (J.Y.); huihongli1998@163.com (H.L.); qskong@mail.hzau.edu.cn (Q.K.); 15377630117@163.com (W.W.); 2 USDA-ARS Vegetable Crops Research Unit, Horticulture Department, University of Wisconsin-Madison, Madison, WI 53706, USA; yiqun.weng@wisc.edu; 3 Hubei Key Laboratory of Vegetable Germplasm Enhancement and Genetic Improvement, Institute of Economic Crops, Hubei Academy of Agricultural Sciences, Wuhan 430064, China; yanch@hbaas.com

**Keywords:** bitter gourd, skin texture, fruit ridge, *EPIDERMAL PATTERNING FACTOR 2-like*, marker-assisted breeding

## Abstract

Bitter gourd (*Momordica charantia* L.) is an economically important vegetable and medicinal crop in many Asian countries. Limited work has been conducted in understanding the genetic basis of horticulturally important traits in bitter gourd. Bitter gourd is consumed primarily for its young, immature fruit, and fruit appearance plays an important role in market acceptability. One such trait is the ridges on the fruit skin. In the present study, molecular mapping of a locus underlying fruit ridge continuity was conducted. Genetic analysis in segregating populations, derived from the crosses between two inbred lines Y1 with continuous ridges (CR) and Z-1-4 with discontinuous ridges (DCR), suggested that CR was controlled by a single recessive gene (*cr*). High-throughput genome sequencing of CR and DCR bulks combined with high-resolution genetic mapping in an F_2_ population delimited *cr* into a 108 kb region with 16 predicted genes. Sequence variation analysis and expression profiling supported the *epidermal patterning factor 2-like* (*McEPFL2*) gene as the best candidate of the *cr* locus. A 1 bp deletion in the first exon of *McEPFL2* in Y1 which would result in a truncated *McEPFL2* protein may be the causal polymorphism for the phenotypic difference between Y1 and Z-1-4. The association of this 1 bp deletion with CR was further supported by gDNA sequencing of *McEPFL2* among 31 bitter gourd accessions. This work provides a foundation for understanding the genetic and molecular control of fruit epidermal pattering and development, which also facilitates marker-assisted selection in bitter melon breeding.

## 1. Introduction

Bitter gourd or bitter melon (2*n* = 2*x* = 22; family *Cucurbitaceae*), is an economically important vegetable crop and medicinal plant in many Asian countries. Bitter gourd is consumed mainly for its immature fruit which is attractive because of its characteristic bitter flavor caused by accumulation of cucuriate triterpenoids (cucurbitacins). In many Asia cultures, bitter gourd is often used for medicinal purposes. Indeed, many studies have documented the medicinal value of bitter gourd [1,2]. Bitter gourd originated in Africa and was domesticated in Asia [3]. The divergence between wild and South Asian cultivars was estimated to be about 6000 years ago, followed by the separation of the Southeast Asian cultivars about 800 years ago [4]. Interestingly, unlike in many crops that exhibit the largest phenotypic changes and traces of selection that happened between wild and cultivar groups, the large differences in bitter gourd are observed between two regional cultivar groups suggesting strong diversifying selection during cultivation in different countries [4].

Bitter gourd is a minor crop. Progress in bitter gourd genetic studies for horticulturally important traits has been very limited due to the lack of genetic and genomics resources. However, the recent release of genome assemblies from two varieties (OHB3-1; Dali-11) [4,5,6] has provided a powerful tool to accelerate molecular mapping in bitter gourd. For example, in recent years, several molecular mapping studies have identified quantitative trait loci (QTL) for fruit quality and yield-related traits in bitter gourd [7,8,9,10,11,12]. Such knowledge will greatly accelerate marker-assisted breeding in bitter gourd.

**Figure 1:**
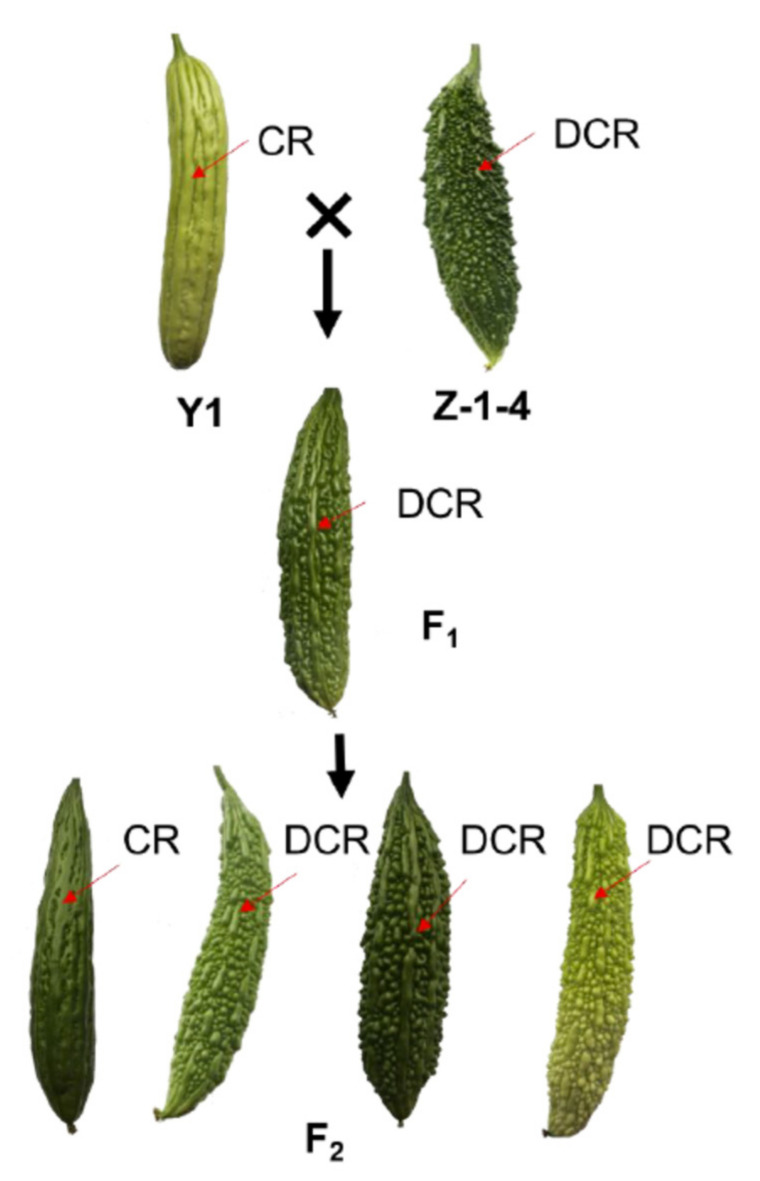
Segregation of ridge patterns on fruit skin in the Y1 × Z-1-4 population showing the recessive inheritance of continuous ridge pattern. CR: continuous ridge; DCR: discontinued ridge.

As in other cucurbit crops, bitter gourd is rich in genetic diversity in terms of fruit shape and surface characters. For example, the bitter gourd skin could be smooth, spiny, warty or bumpy with ridges. For each trait, there may be large variations in the number, size, shape or density, which may reflect consumer preferences in different countries or regions [4,13]. One such trait is the continuity of ridges on fruit surface: some varieties have the fruit ridges stretching from the flower end to the stem end (continuous ridge, or CR), while others may have ridges broken into several pieces (discontinued ridge, DCR) (Figure 1). Fruits with CR are often consumed in south China. Consumers in markets in north China usually prefer fruits with DCR. This type of skin feature is morphologically similar to fruit epidermal features in other cucurbits, such as cucumber (*Cucumis sativus*), which is called ‘ribbing/nonribbing’. In cucumber, fruit ribbing was found to be controlled by a single dominant gene *Fr* [14]. In luffa, *Luffa acutangula* (L.) Roxb, the fruit ridge is also controlled by one major gene with partial dominance [15]. The ‘fruit surface groove’ described in melon (*Cucumis melo* L.) is probably similar to fruit ridge, too; a simply inherited gene encoding an AGAMOUS MADS-box transcription factor was proposed to control this fruit skin feature [16]. Molecular mapping of fruit epidermal feature-related traits in bitter gourd is limited. Several studies investigated fruit tubercles (or warts) and fruit ridges that show simple inheritance [13,17,18,19]. Cui et al. [13] further conducted QTL analysis on fruit wart (fwa) and width of ridge (wr) loci which were both located in the ~1.5 Mbp interval on bitter gourd chromosome 4 (MC4). The molecular mechanism of fruit ridge morphogenesis is not yet clear. Thus, the objective of the present study was to study the genetic basis of fruit ridge patterns in bitter gourd and identify the candidate gene for the simply inherited trait on ridge continuity. We investigated the inheritance of the ridge patterns (CR vs. DCR) in bitter gourd using a segregating population derived from two inbred lines: Z-1-4 (DCR) and Y1 (CR). We conducted BSA-seq and fine mapping for this trait and identified a candidate gene underlying this trait. Knowledge about the genetic basis of fruit appearance trait could facilitate marker-assisted breeding and help understand the molecular mechanisms of the morphogenesis of fruit epidermal features.

## 2. Materials and methods

### 2.1. Plant Materials and Phenotyping

Two inbred lines, Y1 and Z-1-4, were used as parent lines to develop segregating populations. The fruit of Y1 is smooth and covered with continuous ridges (CR), while the fruit of Z-1-4 is covered with heavily pointy warts and discontinuous ridges (DCR). An F_1_ plant was self-pollinated to produce the F_2_ population, which was used to investigate inheritance of ridge patterns on fruit surface. Thirty-one bitter gourd lines, including 18 developed or collected by the senior author’s lab (Wang L.P.) and 13 purchased from the open market in China, were also used to examine the association of ridge patterns and molecular markers.

For phenotyping, in the 2017 growing season, 115 F_2_ plants, their parental lines (15 each) and F_1_ (20) were grown in the plastic greenhouses at the National Center for Vegetable Improvement (Central China) at Huazhong Agricultural University (Wuhan, China). At 15–20 days after pollination (DAP), the fruit skin characters were scored qualitatively as either CR or DCR. To refine map locations, an additional 237 F_2_ plants were phenotyped under the same growth conditions in 2020. Phenotypic data for ridge patterns of 31 bitter gourd varieties were collected in a 2021 plastic house experiment.

### 2.2. Bulked Segregant Analysis (BSA) and Linkage Mapping of cr Locus

BSA was used to identify the subchromosomal location of the gene controlling the ridge pattern variation. Genomic DNAs from the two parental lines and F_2_ plants were extracted with the CTAB method. Two DNA bulks, CR and DCR, were constructed by pooling an equivalent amount of DNA from 20 CR and DCR F_2_ plants, respectively. DNA of CR bulk, DCR bulk, Z-1-4 and Y1 were subjected to high-throughput whole genome sequencing using the HiSeq2500 platform with commercial service (Novogene, Beijing, China).

The quality of resequencing reads was evaluated as described in Yan et al. (2013). High-quality filtered reads were aligned to the draft genomes of bitter gourd cultivar OHB3-1 (ASM199503v1) [5] by the Hisat2 pipeline [20]). SAMtools was used for the variant calling of SNPs and small InDels [21]. The effects of SNP and InDels were estimated with SnpEff [22]. Delta SNP index, or Δ(SNP-index), was calculated by subtracting the SNP index of the DCR pool from that of the CR pool. SNP analysis and sliding-window analysis (window size of 200 kb with a step of 50 kp) was also performed based on the Dali-11 bitter gourd draft genome [6] and the long-read genome of OHB3-1 [4] to establish the marker-trait association. Confidence intervals (CIs) of 95% and 99% were computed for Δ(SNP-index) as described in Tang et al. [23].

For marker analysis, SNPs and InDels in the target region were identified from resequencing reads. SNP genotyping was performed with CAPS assay. Primers for these markers were designed using Primer3plus (https://www.bioinformatics.nl/cgi-bin/primer3plus/, accessed on 1 March 2018). Information about all primers used in this study is provided in Supplementary Table S1.

For initial mapping of the *cr* locus, all markers were first verified for polymorphisms between two parental lines, then applied to 115 F_2_ plants. Refinement of mapping location of the *cr* gene was conducted using an additional 237 F_2_ individuals. Linkage analysis was performed with the JoinMap4 program [24].

### 2.3. Sequence Analysis of the Candidate Gene McEPFL2

Our work suggested that the Arabidopsis homolog *epidermal patterning factor 2-like* (*McEPFL2*; *gene3927*) is a candidate gene for the *cr* locus. We cloned the genomic DNA (gDNA) sequences including part of its flanking sequences from Y1, Z-1-4 and 31 bitter gourd accessions with PCR (see Table S1 for primer information) and Sanger sequencing. Sequence assembly and alignment were performed with Geneious (https://www.geneious.com/, accessed on 1 December 2020).

Total RNA was extracted from peels of Y1 and Z-1-4 ovaries on the day of anthesis using TRIzol reagent following the manufacturer’s protocol. First-strand cDNA was synthesized using the Prime-Script™ RT Reagent Kit (RR037A) with gDNA Eraser (TaKaRa, Kusatsu, Japan). Full-length cDNA *McEPFL2* were cloned from the two parental lines with PCR and Sanger sequencing. Alignment of CDS and deduced amino acid sequences between Y1 and Z-1-4 were carried out with Clustal W. Open reading frame (ORF) prediction was carried out for the predicted CDS sequences by ORFfinder (https://www.ncbi.nlm.nih.gov/orffinder/, accessed on 1 December 2020). The deduced protein sequence was analyzed by SignalP-5 (https://services.healthtech.dtu.dk/, accessed on 1 December 2020) (Almagro Armenteros et al., 2019) to predict the possible signal sequence.

### 2.4. Gene Expression Analysis

We examined the transcriptomes of the peels of the ovaries from two parental lines (on the day of anthesis) with RAN-Seq. Total RNA and mRNA preparations were the same as described above. High-throughput transcriptome sequencing was performed using the Illumina HiSeq4000 platform with a commercial service (Majorbio, Shanghai, China). RNA-seq data were analyzed using the online Majorbio Cloud Platform (www.majorbio.com, accessed on 1 June 2018) [25]. The quality of resequencing reads was evaluated using SeqPrep and sickle. Clean reads were aligned to the genomes OHB3-1 (ASM199503v1) by the Hisat2. Gene expression levels were calculated using featureCounts. Differential expression analysis was carried out by DEGseq [26].

The expression of the candidate gene *McEPFL2* in the peels of the ovaries in Z-1-4 and Y1 was also examined by real-time quantitative reverse transcription (qRT-PCR). The *cyclophilin 2* (*CYP2*) was used as the internal reference. The relative expression level was calculated with the formula 2^−ΔΔCt^. There were three biological replicates and three technical replications for each sample. The significant differences between two samples were estimated using Student’s *t*-test.

### 2.5. Phylogenetic Analysis

We conducted a phylogenetic analysis of *McEPFL2* genes in cucurbits. The amino acid sequence of bitter melon McEPFL2 protein was used as a query to identify its homologs in the National Center for Biological Information (NCBI) by BLASTp. Sequences of *McEPFL2* homologs in melon (*Cucumis melo*), watermelon (*Citrullus lanatus*) and chayote (*Sechium edule*) were downloaded from the cucurbit genome database (https://www.cucurbitgenomics.org/, accessed on 1 March 2022). Nine homologs, including *McEPFL2*, were used to construct a maximum-likelihood (ML) phylogenetic tree in MEGA 11 [27] with the tobacco (*Nicotiana attenuate*) homolog as the outgroup. The ML phylogenetic analyses used the Jones Taylor Thornton (JTT) + γ Distributed (G) model. Node support was evaluated using 1000 bootstrap replicates.

## 3. Results

### 3.1. Continued Ridge of Fruit Skin Is Controlled by a Recessive Gene (cr) in Bitter Gourd

In all greenhouse experiments, the fruits of the parental lines Y1 and Z-1-4 consistently had continued ridge (CR) and discontinued ridge (DCR), respectively (Figure 1). Fruits of reciprocal F_1_ had DCRs. Among the 115 F_2_ plants planted in 2017, 81 and 34 had fruit with DCR and CR, respectively, which is consistent with the 3 DCR: 1 CR segregation ratio (χ^2^ = 1.05, *P* = 0.2582 in χ^2^ test) suggesting CR is recessive to DCR. Among 237 F_2_ plants planted in 2019, 174 and 63 had fruit with DCR and CR, respectively, which is also consistent with the 3 DCR: 1 CR segregation ratio (χ^2^ = 0.28, *P* = 0.5737 in χ^2^ test) suggesting CR is recessive to DCR. Thus, the corresponding gene was designated as *cr* (for *continued ridges*) hereinafter.

### 3.2. The cr Locus Is Fine Mapped in a 108 kb Region on Bitter Melon Chr4

**Figure 2:**
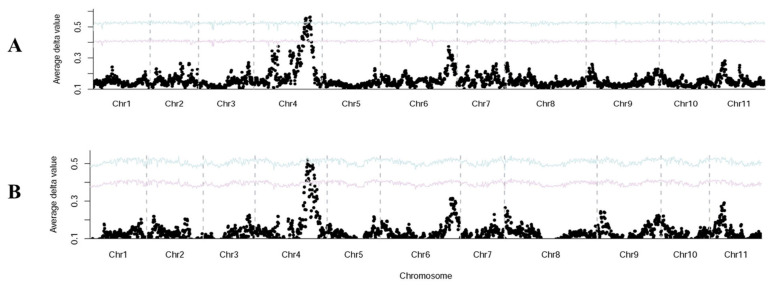
Genomewide ΔSNP-index plot using SNPs between CR and DCR pools suggests that *cr* is located on Chr4 (BLBB01000004.1) in long-read genome of OHB3-1 (**A**) and Chr4 (MC04) in genome Dali-11 (**B**). The Confidence intervals were revealed with purple lines (*p* < 0.05) and blue lines (*p* < 0.01).

High-throughput Illumina whole genome resequencing of the two parent lines (Z-1-4 and Y1) and two bulks (CR and DCR) were carried out, which generated 9.04 million (2.71G), 11.58 million (3.48G), 30.60 million (9.18G) and 32.15 million (9.65G) clean reads, respectively. Main statistics are provided in Supplementary Table S2. SNP calling was performed through a DNA-BSA-pipeline using OHB3-1 (ASM199503v1), as the reference contained only scaffolds but not chromosome-level assemblies [5]. Δ(SNP index) was calculated between two bulks, and 100 SNPs with the highest Δ(SNP index) values at a depth threshold ≥20 were identified, which were provided in Supplementary Table S3. Among them, 31, 55 and 6 loci were from scaffolds NW_019104493.1, NW_019104505.1 and NW_019104528.1, respectively. It turned out that the three scaffolds were located in a region of bitter melon chromosome 4 (Chr4) in the updated draft genome assembly of OHB3-1 [4] and the genome assembly of Dali-11 [6]. Indeed, we conducted SNP calling of the resequencing reads of the two OHB3-1 and Dali-11. The genome-wide Δ(SNP index) graphs are shown in Figure 2A,B, respectively. In both genomes, a single Δ(SNP index) peak was present on Chr4, which was consistent with single locus *cr* controlling the ridge pattern in this population.

**Figure 3:**
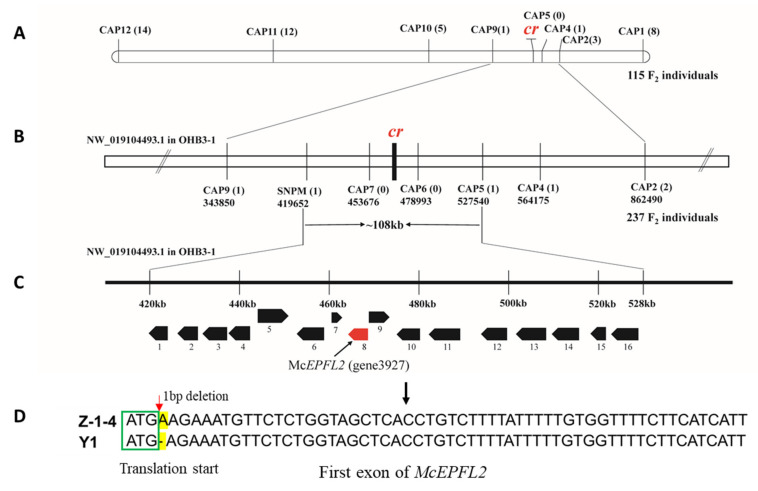
Fine genetic mapping of *cr* locus. (**A**) Initial mapping in 115 F2 plants placed the *cr* locus in a ~210 kb region on scaffold NW_019104493.1 in OHB3-1. Numbers in brackets after the name of markers are recombinants. The *cr* candidate region is further narrowed down to 108 kb region (**B**) that harbors 16 predicted genes (**C**). Gene # 8 (red) encodes the EPIDERMAL PATTERNING FACTOR 2 protein (*McEPFL2*), which is the best candidate for *cr*. A 1 bp deletion is present in the first exon of *McEPFL2* of the inbred line Y1 with CR (**D**).

To map the *cr* locus, eight CAPS markers were designed based on the SNPs located in scaffolds NW_019104493 or NW_019104505.1 (Tables S1 and S3), which were used in CAPS assays in 115 F_2_ plants (Supplementary Table S1). Linkage analysis suggested that *cr* is located in a region between two markers, CAP4 and CAP9, which were physically ~220kb apart whereas CAP5 was cosegregating with the *cr* locus (Figure 3A).

To refine the map locations of the *cr* locus, we developed two new markers (CAP6 and CAP7) in the 220 kb region, which, together with the other five markers, were used to genotype 237 additional F_2_ individuals. Then, the *cr* locus was delimited between SNPM and CAP5 with one recombinant on each side, and CAP6 and CAP7 were cosegregating with *cr* (Figure 3B). This allowed us to narrow down the candidate gene into a 108 kb region.

### 3.3. Identification and Characterization of cr Candidate Gene

According to the genome OHB3-1 (ASM199503v1), 16 genes were predicted in the 108 kb region (Figure 3C), which are listed in Supplementary Table S4. DNA sequence variation in this region between the two parental lines was examined. In total, 83 SNPs or small InDels were identified and further annotated by snpEFF. The detailed information of these variants and summary statistics are provided in Supplementary Tables S5 and S6, respectively. Among them, 61 sequence variants occurred in downstream or upstream regions, 11 in 5′ UTR regions and 1 in intronic region. In exonic regions, 10 variants were found, of which 9 SNPs were annotated to be synonymous variation and one had a 1 bp InDel (Pos: 465777) that suggested to be frameshift variation (Tables S5 and S6).

The 1 bp InDel at position 465,778 of the OHB3-1 scaffold NW_019104493.1 was located in the first exon of *gene3927*, which was predicted to encode the EPIDERMAL PATTERNING FACTOR 2-like protein (McEPFL2). The genomic DNA of *McEPFL2* was 2242 bp in length with three exons and two introns. Its full-length cDNA and coding sequence (CDS) were 1879 bp and 357 bp, respectively.

The genomic DNA (gDNA) sequences of *McEPFL2*, including part of its flanking sequences, from the two parental lines (Y1 and Z-1-4) were cloned using gene-specific primers (Table S1). Alignment of gDNA sequences between them (Supplementary Figure S1A) clearly shows that the 1 bp InDel variation described above was the only sequence variation inside this region between the two parental lines. Partial cDNA sequences of *McEPFL2*, including the complete coding sequence (CDS), were also cloned from the two parental lines, which confirmed the presence of 1 bp deletion in in the first exon of *McEPFL2* in Y1 (*crcr*) (Figure 3D and Figure S1B). The 1 bp deletion in Y1 would lead to a frameshift mutation and shortened amino acid residues in the deduced protein (Supplementary Figure S1C).

### 3.4. Allelic Variation of McEPFL2 in Natural Bitter Gourd Populations

To further verify the association of the 1 bp deletion inside the first exon of *McEPFL2* gene with ridge patterns in bitter gourd, we cloned the gDNA sequences of *McEPFL2* from 31 additional accessions. Representative fruit images of the 31 lines are shown in Supplementary Figure S2. Among them, 10 had CR and 21 had DCR. Alignment of DNA sequences around the target locus from 31 lines are illustrated in Supplementary Figure S3 in which the ridge patterns of these lines are also included. Among them, 29 lines were homozygous at the target locus; all 10 CR lines carried the same 1 bp deletion as in Y1 while 19 DCR lines carried the alternate allele (no deletion) as found in Z-1-4. Two DCR lines (A8 and A13) were heterozygous at this locus (Figure S3B). These observations provided convincing evidence that *McEPFL2* was the candidate gene for the *cr* locus, and the 1 bp deletion may be the causal polymorphism associated with the CR and DCR ridge pattern variation in bitter gourd. In addition, the DCR phenotype of the two heterozygous lines also supported the recessive nature of CR.

### 3.5. Expression of cr Candidate Gene McEPFL2

**Figure 4:**
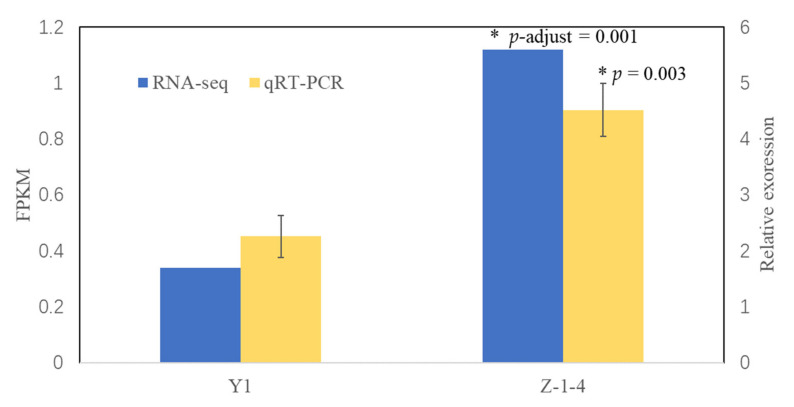
Expression analysis of *McEPFL2* in fruit epidermal tissues in Z-1-4 and Y1 by RNA-seq (blue) and qRT-PCR (yellow). Error bars are mean ± standard error. Significance of expression level was made between the two genotypes in each experiment with *t*-tests. Asterisks (*) means *p* < 0.05 by student’s *t*-test.

To further prove the candidacy of *McEPFL2*, we examined its expression pattern in the fruit skin of Z-1-4 and Y1 using qRT-PCR. The result is illustrated in Figure 4. There was a higher expression of *McEPFL2* in Z-1-4 with DCR. We have also conducted transcriptome profiling of the fruit peels through RNA-Seq of the Z-1-4 and Y1 fruit peels (see Table S2 for RNA-Seq statistics). We calculated the log2 fold changes of the 16 genes inside the *cr* candidate region. Relevant statistics of these genes are provided in Supplementary Table S7. Using the thresholds of *p*-adjust < 0.05 and | log2(Fold Change) | > = 1, three genes (*gene3923*, *gene3927* and *gene3929*) showed differential expression in fruit epidermal tissues between two parent lines. Consistent with qRT-PCR, the expression of *McEPFL2* was lower in Y1 than that in Z-1-4 (*p* = 0.003). The sequence variants (SNPs) inside *gene3923* and *gene3929* were all synonymous mutations (Table S5). These data provided further evidence to support *McEPFL2* as the candidate gene of the *Cr* locus.

### 3.6. Phylogenetic Analysis

The amino acid sequences of McEPFL2 from bitter melon and nine other cucurbits in the *Cucurbitaceae* family (provided in Supplementary Table S8) were used to construct a maximum-likelihood (ML) phylogenetic tree, which is presented in Supplementary Figure S4. Among these nine sequences, the first divergent species appears to be bitter gourd, followed by chayote. Sequences from four species in *Benicaseae* formed a group. Sequences from three genus in *Cucurbiteae* formed a group. Species belonging to *Benicaseae* and *Cucurbiteae* formed the sister clade. These results were similar with hypothesis of the evolutionary history of the taxa [28].

## 4. Discussion

The morphology of the fruit ridge in bitter gourd is an important trait which may not only affect consumer preferences, but also be related to performance in cleaning, packing and transportation. For breeders, it is important to breed varieties with certain fruit appearance to meet the requirements of targeted markets. In this study, we investigated the genetic basis of a fruit appearance quality-related trait, ‘continued ridges’, and found it was controlled by a simply inherited recessive locus, *cr* (Figure 1). Through combined analysis of BSA-Seq and fine genetic mapping, *cr* was mapped to a ~108 kb region on bitter gourd chromosome 4 (MC04) with 16 annotated genes (Figure 2 and Figure 3; Table S4). Multiple lines of evidence supported *McEPFL2* as the best candidate for the *Cr* locus. First, we examined sequence variation of the 16 genes between the two parental liens and found that *McEPFL2* was the only gene carrying a nonsynonymous mutation (1 bp deletion in Y1) (Tables S5 and S6). Second, in the transcriptomes of fruit peels of Y1 and Z-1-4 from RNA-Seq, *McEPFL2* exhibited differential expression between the two lines, with Y1 (CR) having lower expression than in Z-1-4 (DCR) (Table S7), which was confirmed by qRT-PCR (Figure 4). Lastly, we cloned the gDNA sequences surrounding the 1 bp deletion among 31 bitter gourd lines and found complete association of the skin ridge patterns with the alleles at the 1 bp deletion locus (Figure S3).

To our knowledge, the present work was the first description of skin ridge continuity trait (CR vs. DCR) in bitter gourd. Cui et al. [13] previously described two relevant fruit skin traits in bitter gourd: *Fwa* (fruit wart) and *Wr* (width of ridge). Bitter gourd fruit may be warty or smooth. There is also variation in the width of fruit ridges. Through QTL mapping, Cui et al. [13] identified a major-effect QTL, *Fwa/Wr*, underlying both warty fruit and ridge width that could explain 17.7–56.7% observed variance, suggesting a major locus for the two traits. This QTL was located in ~1.5 Mbp interval MC04 with ~218 predicted genes (genome Dali) [13]. We compared the physical locations on MC4 of the *Fwa/Wr* and *Cr* overlapped, suggesting that the same gene may be governing fruit wart, width of ridge and continue ridges. Warts are distributed between ridges on the fruits. We observed that fruits with CR usually have fewer warts (even nonwarty) and wider ridges than those with DCR. Thus, *Fwa*, *Wr* and *Cr* in bitter gourd are actually controlled by the same locus. However, further investigation is needed to confirm this.

The fruit ribs in cucumber [14] are very similar to ridges described in the present study. Miao et al. [14] found that ribbing in cucumber is dominant to nonribbing that is controlled by a single locus, *Fr*, which was mapped on cucumber Chr5. The gene underlying fruit ribbing in cucumber is unknown. We searched the cucumber genome assemblies (9930v3.0 and Gy14v2.1; https://cucurbitgenomcs.org/, accessed on 1 April 2022) and there were 13 genes annotated as “Epidermal patterning factor-like protein”, but none of them were located on cucumber Chr5. Similarly, the single gene (*Cmfsg*) for the fruit surface groove of melon was proposed to encode an AGAMOUS MADS-box transcription factor [16]. These observations suggest that the cucumber ribbing/nonribbing or melon fruit groove may have different genetic bases from fruit ridge continuity (CR/DCR) in bitter gourd.

Our work supports *McEPFL2* as the most possible candidate for the *Cr* locus in bitter gourd. In Arabidopsis, EPFL2 is a member of the cysteine-rich secreted peptide of the EPF/EPFL (epidermal patterning factor/EPF-like) gene family. This gene family encodes plant-specific secretory peptides that are involved in various developmental pathways, including epidermal cell patterning, inflorescence architecture and lateral shoot organ patterning [29,30,31,32]. Hunt and Gary [33] found that Arabidopsis *EPF2* controls asymmetric cell divisions during stomatal development and controls stomatal density. Hara et al. [34] further showed that *EPF2* limits the final density of epidermal cells by acting as a key component of a negative feedback loop that limits the number of cells entering the stomatal lineage. More recent studies show that Arabidopsis *EPFL2* is specifically expressed in the boundary domains of various shoot organs and related to regulation of shoot meristem size [32], cotyledon growth [35], leaf and ovule positioning [36]. There is no report on the involvement of *EPFL2* in fruit epidermis morphogenesis, as we observed, in affecting the ridge continuity in bitter gourd. Thus, our finding in this work may represent a novel role for the *EPFL2* protein.

In the DCR parental line Z-1-4, the open reading frame (ORF) of *McEPFL2* was 357 bp that encodes a peptide with 118 amino acid residues that contains a hydrophobic amino acid sequence at the N-terminal (Figure S1C). For Y1, the 1 bp deletion inside the first exon would lead a frame shift mutation and the ORF of 294 bp, which encodes a peptide with 97 amino acid residues (Figure S1C) which was predicted to lose its N-terminal signal peptide sequence (Sec/SPI). How this affects the function *McEPFL2* and the different skin ridge patterns merits further investigation.

## Data Availability

All data for the reported work have been provided in the manuscript or in the Supplementary Materials.

## Supplementary Materials

The following supporting information can be downloaded at: https://www.mdpi.com/article/10.3390/genes13071148/s1, Figure S1: Alignment of genomic DAN (A), partial cDAN (B) and deduced amino acid (C) sequences of the *McEPFL2* gene; Figure S2: Fruit ridge patterns of 18 bitter melon inbred lines (A) and 13 commercial varieties (B) used in the present study; Figure S3: Alignment of sequences of the first exon of the Mc*EPFL2* gene among 31 bitter melon varieties; Figure S4: The maximum-likelihood (ML) phylogenetic tree of the EPFL2 family members in Cucurbitaceae family, including *Cucumis sativus* (cucumber), *Cucumis melo* (melon), *Benincasa hispida* (Wax gourd), *Citrullus lanatus* (water melon), *Cucurbita maxima*, *Cucurbita pepo*, *Cucurbita moschata*, *Sechium edule* (Chayote), and *Momordica charantia* (Bitter gourd, red); Table S1: Information of primers used in this study; Table S2: Statistics of sequencing date; Table S3: List of 100 SNPs with the highest Δ(SNP-index) values between CR and DCR pools; Table S4: Predicted genes in the *cr* candidate gene region between molecular markers SNPM and CAP5; Table S5: Annotation of SNP and INDEL by snpEFF; Table S6: Statistics of variants annotated with different mutant types; Table S7: Expression of genes in the *cr* candidate gene region in Z-1-4 and Y1 by RNA-seq (Y1 vs. Z-1-4); Table S8: Protein sequences of *McEPFL2* homologs from 10 plant species.
