# Supplementary material for: Epidermal Patterning Factor 2-like (McEPFL2): A Putative Candidate for the Continuous Ridge (cr) Fruit Skin Locus in Bitter Gourd (Momordica charantia L.)
Source: Genes (Basel). 2022 Jun 25;13(7):1148. doi: 10.3390/genes13071148 (PMC9316824; doi:10.3390/genes13071148)
Supplement: Supplementary file 1 [file genes-13-01148-s001.zip › genes-1783079-supplementary.pdf]

Figure S1. Alignment of genomic DAN (A), partial cDAN (B) and deduced amino acid (C) sequences of the *McEPFL2* gene. The 1 bp indel in both gDNA and cDNA is highlighted in red.

A. Alignment of genomic sequences including the promoter region between Z-1-4 and Y1.

```
Z-1-4_gDNA      CTTGCCAAATCATGTGATCCTTCAAACCATGGCCGTGGATCATGTGAAGTGCAACAGATG 60
Y1_gDNA         CTTGCCAAATCATGTGATCCTTCAAACCATGGCCGTGGATCATGTGAAGTGCAACAGATG 60
                ************************************************************

Z-1-4_gDNA      TACCTCTTCCTACATTAAAAACTAACTCTATATAAAATGCTGGCAACATACTAAATCATC 120
Y1_gDNA         TACCTCTTCCTACATTAAAAACTAACTCTATATAAAATGCTGGCAACATACTAAATCATC 120
                ************************************************************

Z-1-4_gDNA      TGTCACCAACATTTCCAACAAACCTTGCAGTCTGTTTTCTCCTTTTGCTCTACCACTTCA 180
Y1_gDNA         TGTCACCAACATTTCCAACAAACCTTGCAGTCTGTTTTCTCCTTTTGCTCTACCACTTCA 180
                ************************************************************

Z-1-4_gDNA      TTCTTCCAGGCTTCTGCATTATCAAAAAACAATGAAGAAATGTTCTCTGGTAGCTCACCT 240
Y1_gDNA         TTCTTCCAGGCTTCTGCATTATCAAAAAACAATG-AGAAATGTTCTCTGGTAGCTCACCT 239
                ********************************** *************************

Z-1-4_gDNA      GTCTTTTATTTTTGTGGTTTTCTTCATCATTCTAATGATTGGCAGAAGTCTTGATGCAAC 300
Y1_gDNA         GTCTTTTATTTTTGTGGTTTTCTTCATCATTCTAATGATTGGCAGAAGTCTTGATGCAAC 299
                ************************************************************

Z-1-4_gDNA      ATCTCGCTGGGAACACAGTAAGAAAACTCATCATCTATGCGGTTTACATTGTCTACTTAT 360
Y1_gDNA         ATCTCGCTGGGAACACAGTAAGAAAACTCATCATCTATGCGGTTTACATTGTCTACTTAT 359
                ************************************************************

Z-1-4_gDNA      TTTGATATTCTAGTTGCGGAAGATATGCATCTGTATCCCTATAACGGATACACCGACATT 420
Y1_gDNA         TTTGATATTCTAGTTGCGGAAGATATGCATCTGTATCCCTATAACGGATACACCGACATT 419
                ************************************************************

Z-1-4_gDNA      TTTCTCTGATTTAATCAGTTATGACATCTAAGCCTTTCAAGTGTGTGATATTTCAGTGAG 480
Y1_gDNA         TTTCTCTGATTTAATCAGTTATGACATCTAAGCCTTTCAAGTGTGTGATATTTCAGTGAG 479
                ************************************************************

Z-1-4_gDNA      CTTTAATGCAGAAGATATTCACAGAACAGTGGACAGTTCAATGCACCAGAAGGTATTATG 540
Y1_gDNA         CTTTAATGCAGAAGATATTCACAGAACAGTGGACAGTTCAATGCACCAGAAGGTATTATG 539
                ************************************************************

Z-1-4_gDNA      TTACTTTCAGCCCACATTTATCCACCCAGTGTTAAACCTTTTGCGAATAAATAAATAAAG 600
Y1_gDNA         TTACTTTCAGCCCACATTTATCCACCCAGTGTTAAACCTTTTGCGAATAAATAAATAAAG 599
                ************************************************************

Z-1-4_gDNA      GTCACCTAACTGAGACAATAATATGAAAAATATAAATAATAGATGCTCGCTTGAGAGTAC 660
Y1_gDNA         GTCACCTAACTGAGACAATAATATGAAAAATATAAATAATAGATGCTCGCTTGAGAGTAC 659
                ************************************************************

Z-1-4_gDNA      AGTAAACAAGAAAAACCATGGCAACATACTGCAAAAAAGTCCTCATTCAGTTTCGTTATC 720
Y1_gDNA         AGTAAACAAGAAAAACCATGGCAACATACTGCAAAAAAGTCCTCATTCAGTTTCGTTATC 719
                ************************************************************

Z-1-4_gDNA      ATGTGTGACTGTGCAGGAAAGGGCTAAAGAAGTACTTGGTATGGAGTTATACCCTACAGG 780
Y1_gDNA         ATGTGTGACTGTGCAGGAAAGGGCTAAAGAAGTACTTGGTATGGAGTTATACCCTACAGG 779
                ************************************************************

Z-1-4_gDNA      ATCCAGCCTCCCAGACTGCTCCCATGCATGCGGTCCATGTTTTCCATGCAAAAGGGTGAT 840
Y1_gDNA         ATCCAGCCTCCCAGACTGCTCCCATGCATGCGGTCCATGTTTTCCATGCAAAAGGGTGAT 839
                ************************************************************
```

```
Z-1-4_gDNA      GGTGAGCTTCAAGTGCTCTGTTGCAGAGTCCTGTCCAACTGTTTACAGATGCATGTGTAA 900
Y1_gDNA         GGTGAGCTTCAAGTGCTCTGTTGCAGAGTCCTGTCCAACTGTTTACAGATGCATGTGTAA 899
                ************************************************************

Z-1-4_gDNA      AGGGAAATATTACCATGTACCCTCCAATTGAGCAAAAATTCAATCTGGAGGAATTTGCAG 960
Y1_gDNA         AGGGAAATATTACCATGTACCCTCCAATTGAGCAAAAATTCAATCTGGAGGAATTTGCAG 959
                ************************************************************

Z-1-4_gDNA      CAAAGAACAGAGAAATTAATGATTGGGTTGGCACTTAGCACTTATATGATGACTAAGGAT 1020
Y1_gDNA         CAAAGAACAGAGAAATTAATGATTGGGTTGGCACTTAGCACTTATATGATGACTAAGGAT 1019
                ************************************************************

Z-1-4_gDNA      ATAACATAGACTAAGACTATAGTCTCTATTGGTTTCATTAGGGTTTATTTTCTTCTACCG 1080
Y1_gDNA         ATAACATAGACTAAGACTATAGTCTCTATTGGTTTCATTAGGGTTTATTTTCTTCTACCG 1079
                ************************************************************

Z-1-4_gDNA      GAATCATTTTGTAAGAGAAAATAGCTGAGGGAACAAATTTTCAAGCACGGGAAGTTTAAT 1140
Y1_gDNA         GAATCATTTTGTAAGAGAAAATAGCTGAGGGAACAAATTTTCAAGCACGGGAAGTTTAAT 1139
                ************************************************************

Z-1-4_gDNA      TAGGTAGTGATAACTCAGTTTGGTCTACTCTAGGAGTGTGCAAAATTGTTTCCCTAGTTA 1200
Y1_gDNA         TAGGTAGTGATAACTCAGTTTGGTCTACTCTAGGAGTGTGCAAAATTGTTTCCCTAGTTA 1199
                ************************************************************

Z-1-4_gDNA      TATAACTCTACTTTAAAGACATGTCGTGACATTTGGCTTGTCCTGTTTCTGCCTTCCCAG 1260
Y1_gDNA         TATAACTCTACTTTAAAGACATGTCGTGACATTTGGCTTGTCCTGTTTCTGCCTTCCCAG 1259
                ************************************************************

Z-1-4_gDNA      GTTTCTAAGTTGAGAATATACAGTTGAATGGAGGTTTGGAAAACAAGCCAGAATCAGAGG 1320
Y1_gDNA         GTTTCTAAGTTGAGAATATACAGTTGAATGGAGGTTTGGAAAACAAGCCAGAATCAGAGG 1319
                ************************************************************

Z-1-4_gDNA      ATAACCTTGTAAAGTTTAGTGTGTCAGAGGGACATACATCCATGTTTTCAAATTCAACCA 1380
Y1_gDNA         ATAACCTTGTAAAGTTTAGTGTGTCAGAGGGACATACATCCATGTTTTCAAATTCAACCA 1379
                ************************************************************

Z-1-4_gDNA      AAATACATATTCATTCAGCCCGAGCTCAAGGAACTATGTTCTGCTCTTTAGGTTGAAGGA 1440
Y1_gDNA         AAATACATATTCATTCAGCCCGAGCTCAAGGAACTATGTTCTGCTCTTTAGGTTGAAGGA 1439
                ************************************************************

Z-1-4_gDNA      AGCAATACGGAAAAATTCATAAAAGATATGATCAAAACCAATGAACCAGGATCACTGTTT 1500
Y1_gDNA         AGCAATACGGAAAAATTCATAAAAGATATGATCAAAACCAATGAACCAGGATCACTGTTT 1499
                ************************************************************

Z-1-4_gDNA      TTGTGAATGACCATCTGTAGAAGAGGATTTCCAAACAAAACAATCAATGAGGTAAATATA 1560
Y1_gDNA         TTGTGAATGACCATCTGTAGAAGAGGATTTCCAAACAAAACAATCAATGAGGTAAATATA 1559
                ************************************************************

Z-1-4_gDNA      ACAATAGTAGAGTATCCTATCATTACAAGAGCAAACAACAATGGCTGGTTTGCAGAAATT 1620
Y1_gDNA         ACAATAGTAGAGTATCCTATCATTACAAGAGCAAACAACAATGGCTGGTTTGCAGAAATT 1619
                ************************************************************

Z-1-4_gDNA      AATGAAAATGTAAACTAAGGGAAATTGTAGAAGAGAAAAAAAAGGCATAAAAATAGTGAG 1680
Y1_gDNA         AATGAAAATGTAAACTAAGGGAAATTGTAGAAGAGAAAAAAAAGGCATAAAAATAGTGAG 1679
                ************************************************************

Z-1-4_gDNA      TTTTCTATAGCAAATAACACTTGTAATTCAAACATTCAAACAGAAATTAAACAGCTGCAC 1740
Y1_gDNA         TTTTCTATAGCAAATAACACTTGTAATTCAAACATTCAAACAGAAATTAAACAGCTGCAC 1739
                ************************************************************

Z-1-4_gDNA      GGACCCAAACTGCTAAACAATATGAATTTGAAAACCACCCAAAACTCGTTCTATAACAGA 1800
Y1_gDNA         GGACCCAAACTGCTAAACAATATGAATTTGAAAACCACCCAAAACTCGTTCTATAACAGA 1799
                ************************************************************
```

```
Z-1-4_gDNA      TGGATTCTAGACCTCAAGCTCACTCTTAGGCATGAAAAAAACAAACTTTCTTGAGTCCCC 1860
Y1_gDNA         TGGATTCTAGACCTCAAGCTCACTCTTAGGCATGAAAAAAACAAACTTTCTTGAGTCCCC 1859
                ************************************************************

Z-1-4_gDNA      GACAAAGACATCAGCATACTTCTGCCTTTATCAATGCTCATTCAAATATCACACTCACAC 1920
Y1_gDNA         GACAAAGACATCAGCATACTTCTGCCTTTATCAATGCTCATTCAAATATCACACTCACAC 1919
                ************************************************************

Z-1-4_gDNA      CGAACAGGTTCGTAAATTCTTCATAACCTCAAACCTGGAATCCAGTACCCTATCAAAATT 1980
Y1_gDNA         CGAACAGGTTCGTAAATTCTTCATAACCTCAAACCTGGAATCCAGTACCCTATCAAAATT 1979
                ************************************************************

Z-1-4_gDNA      TCATCCGTTGTTAGTTTCTGATGGTTTCGTCATCGCAGACTATAAATTGCACCCTAATCT 2040
Y1_gDNA         TCATCCGTTGTTAGTTTCTGATGGTTTCGTCATCGCAGACTATAAATTGCACCCTAATCT 2039
                ************************************************************

Z-1-4_gDNA      ATGATATTCTTCTTTCTCGATGAGTGTTCAGAAGTAGGAACTTGGATAACTTAATGCAAT 2100
Y1_gDNA         ATGATATTCTTCTTTCTCGATGAGTGTTCAGAAGTAGGAACTTGGATAACTTAATGCAAT 2099
                ************************************************************

Z-1-4_gDNA      CAGAACCCTAAGACTATCTCATCCACGTTCGTGAATACCATAAGGAACAGGGGAAAATAC 2160
Y1_gDNA         CAGAACCCTAAGACTATCTCATCCACGTTCGTGAATACCATAAGGAACAGGGGAAAATAC 2159
                ************************************************************

Z-1-4_gDNA      TCCGCAGGAATCATATGTACCATTATAGCAGATTTCGTGTTGATATCGAAATTCAATAGG 2220
Y1_gDNA         TCCGCAGGAATCATATGTACCATTATAGCAGATTTCGTGTTGATATCGAAATTCAATAGG 2219
                ************************************************************

Z-1-4_gDNA      AAATCCACAGACTAACCGAACCAGAATGAGAACAGACAAATTGATGGATGGAAATATTCT 2280
Y1_gDNA         AAATCCACAGACTAACCGAACCAGAATGAGAACAGACAAATTGATGGATGGAAATATTCT 2279
                ************************************************************

Z-1-4_gDNA      GTGAGAAATTTCCATGTAATTATGGCCGATTATCTAGCGAGATTAATTCCAGACAACCCC 2340
Y1_gDNA         GTGAGAAATTTCCATGTAATTATGGCCGATTATCTAGCGAGATTAATTCCAGACAACCCC 2339
                ************************************************************

Z-1-4_gDNA      GAAACATGTC          2350
Y1_gDNA         GAAACATGTC          2349
                **********
```

B. Alignment of partial cDNA sequences between Z-1-4 and Y1.

```
Z-1-4_cDNA      TCATTCTTCCAGGCTTCTGCATTATCAAAAAAACAATGAAGAAATGTTCTCTGGTAGCTCA 60
Y1_cDNA         TCATTCTTCCAGGCTTCTGCATTATCAAAAAAACAATGA-GAAATGTTCTCTGGTAGCTCA 59
                *************************************** *********************

Z-1-4_cDNA      CCTGTCTTTTATTTTTGTGGTTTTCTTCATCATTCTAATGATTGGCAGAAGTCTTGATGC 120
Y1_cDNA         CCTGTCTTTTATTTTTGTGGTTTTCTTCATCATTCTAATGATTGGCAGAAGTCTTGATGC 119
                ************************************************************

Z-1-4_cDNA      AACATCTCGCTGGGAACACATGAGCTTTAATGCAGAAGATATTCACAGAACAGTGGACAG 180
Y1_cDNA         AACATCTCGCTGGGAACACATGAGCTTTAATGCAGAAGATATTCACAGAACAGTGGACAG 179
                ************************************************************

Z-1-4_cDNA      TTCAATGCACCAGAAGGAAAGGGCTAAAGAAGTACTTGGTATGGAGTTATACCCTACAGG 240
Y1_cDNA         TTCAATGCACCAGAAGGAAAGGGCTAAAGAAGTACTTGGTATGGAGTTATACCCTACAGG 239
                ************************************************************

Z-1-4_cDNA      ATCCAGCCTCCCAGACTGCTCCCATGCATGCGGTCCATGTTTTCCATGCAAAAGGGTGAT 300
Y1_cDNA         ATCCAGCCTCCCAGACTGCTCCCATGCATGCGGTCCATGTTTTCCATGCAAAAGGGTGAT 299
                ************************************************************
```

```
Z-1-4_cDNA      GGTGAGCTTCAAGTGCTCTGTTGCAGAGTCCTGTCCAACTGTTTACAGATGCATGTGTAA 360
Y1_cDNA         GGTGAGCTTCAAGTGCTCTGTTGCAGAGTCCTGTCCAACTGTTTACAGATGCATGTGTAA 359
                ************************************************************

Z-1-4_cDNA      AGGGAAATATTACCATGTACCCTCCAATTGAGCAAAAATTCAATCTGGAGGAATTTGCAG 420
Y1_cDNA         AGGGAAATATTACCATGTACCCTCCAATTGAGCAAAAATTCAATCTGGAGGAATTTGCAG 419
                ************************************************************

Z-1-4_cDNA      CAAAGAACAGAGAAATTAATGATTGGGTTGGCACTTAGCACT  462
Y1_cDNA         CAAAGAACAGAGAAATTAATGATTGGGTTGGCACTTAGCACT  461
                ******************************************
```

C. Alignment of deduced amino acid sequences between Z-1-4 and Y1.

```
Z-1-4      MKKCSLVAHLSFIFVVFFIILMIGRSLDATSRWEHMSFNAEDIHRTVDSSMHQKERAKEV     60
Y1         ---------------------MIGRSLDATSRWEHMSFNAEDIHRTVDSSMHQKERAKEV     39
                                ***************************************

Z-1-4      LGMELYPTGSSLPDCSHACGPCFPCKRVMVSFKCSVAESCPTVYRCMCKGKYYHVPSN       118
Y1         LGMELYPTGSSLPDCSHACGPCFPCKRVMVSFKCSVAESCPTVYRCMCKGKYYHVPSN       97
           **********************************************************
```

Figure S2 Fruit ridge patterns of 18 bitter gourd inbred lines (A) and 13 commercial varieties (B) used in the present study. CR: continue ridge; DCR: discontinued ridge.

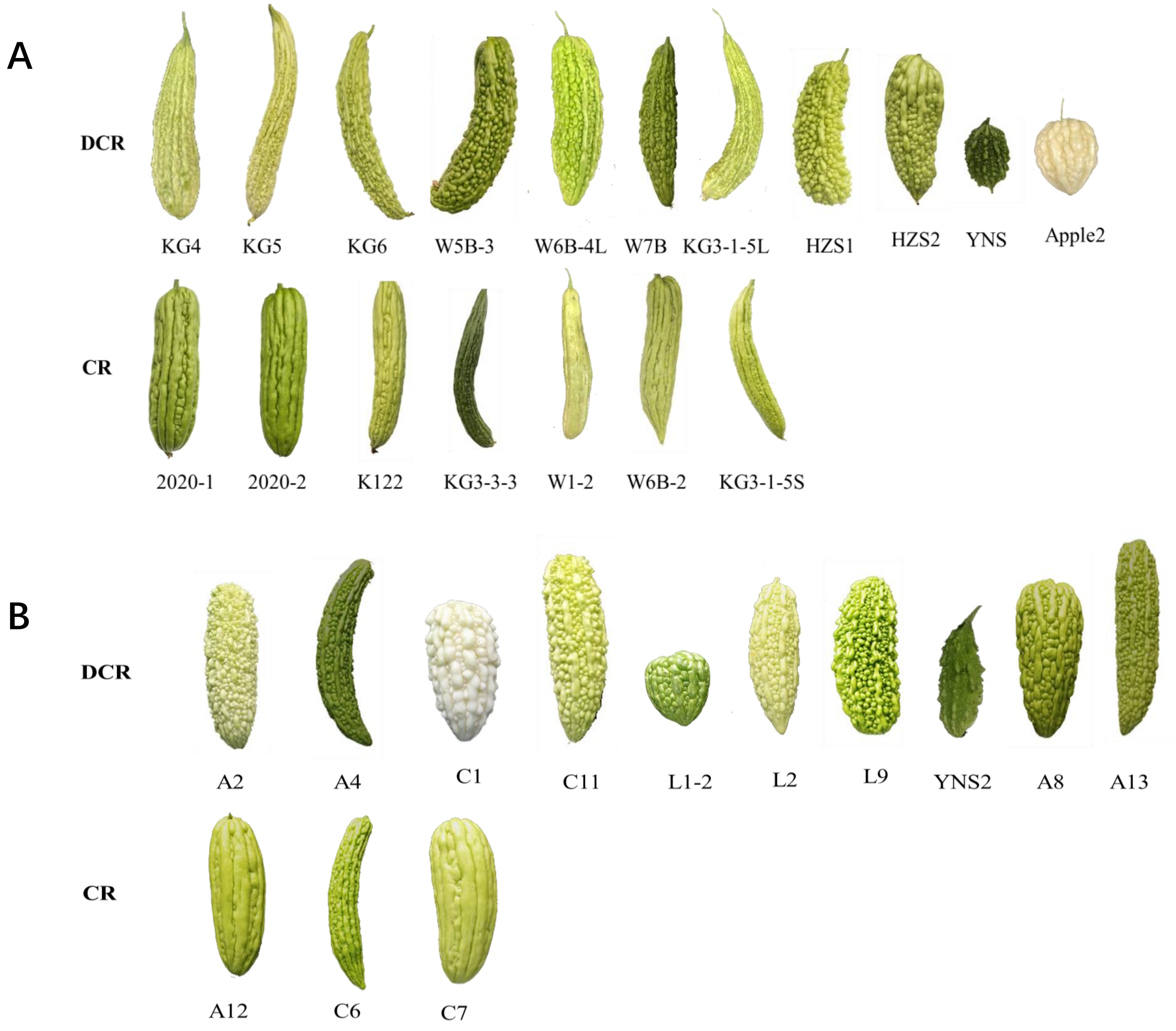

Figure S3 Alignment of sequences of the first exon of the Mc*EPFL2* gene among 31 bitter gourd varieties. A. All 10 CR varieties carried the 1 bp deletion while all 19 DCR lines carry the same allele as the reference (DCR). B. Two DCR lines are heterozygous at the locus.

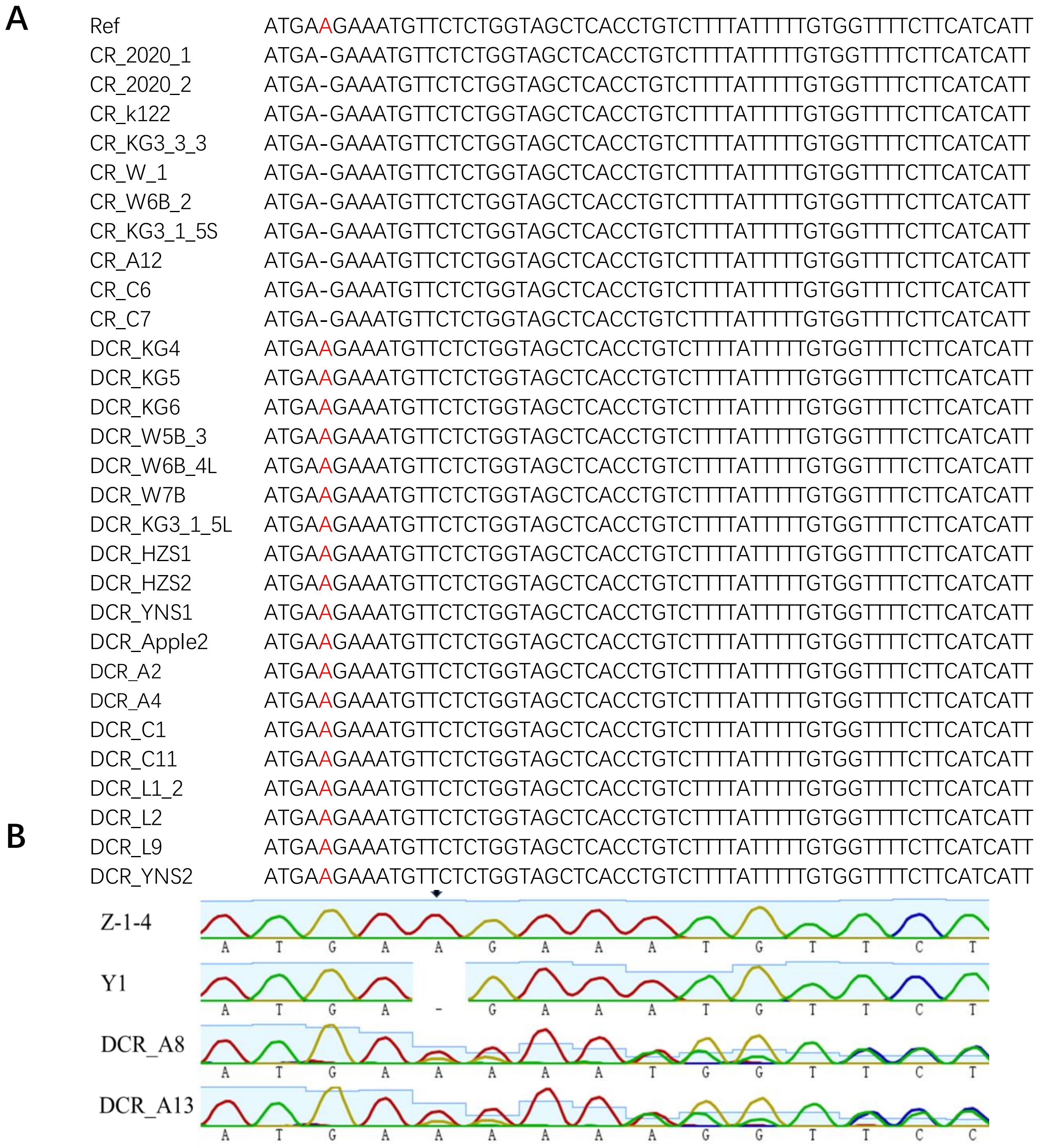

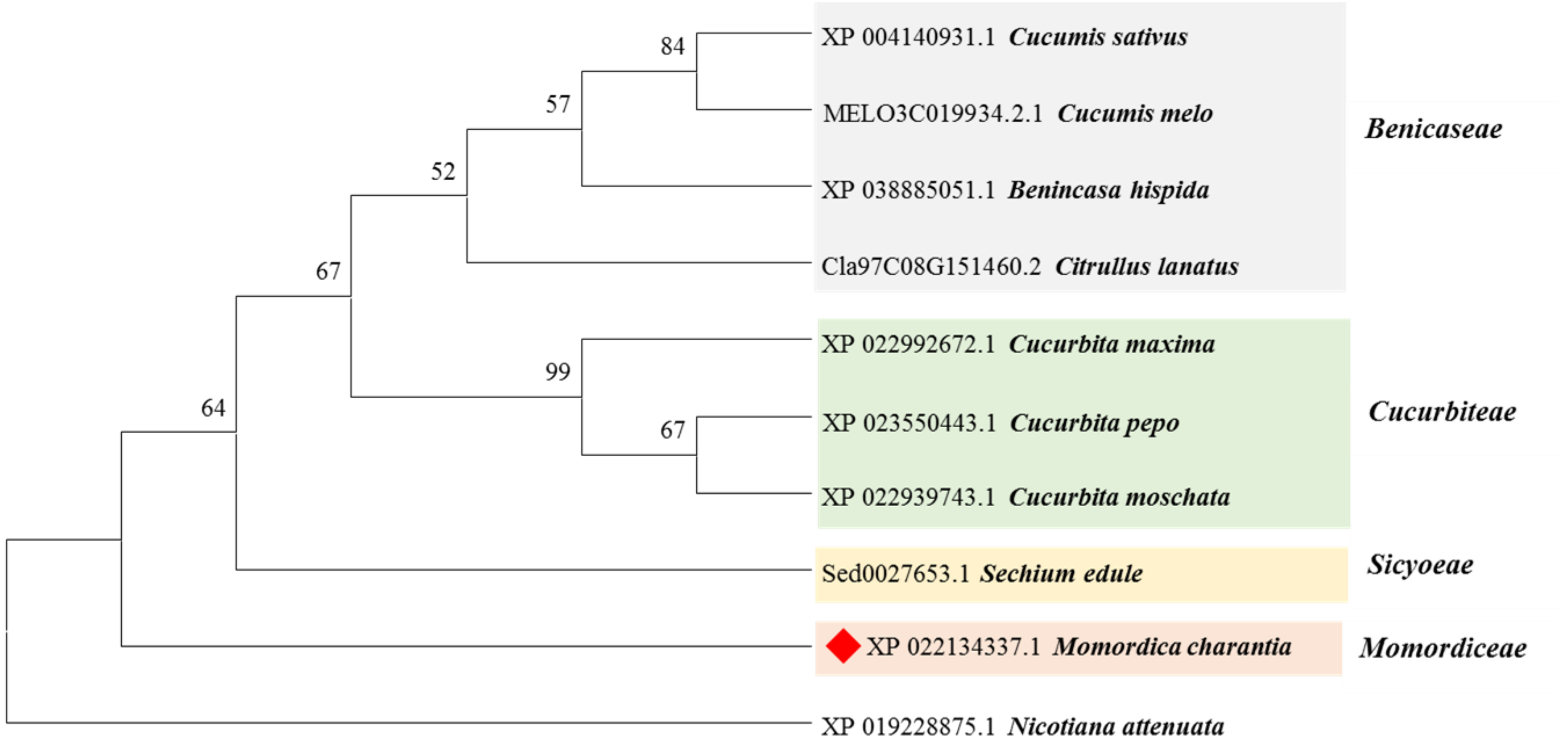

Figure S4 The maximum-likelihood（ML）phylogenetic tree of the EPFL2 family members in Cucurbitaceae family, including *Cucumis sativus*(cucumber), *Cucumis melo* (melon), *Benincasa hispida* (Wax gourd), *Citrullus lanatus* (water melon), *Cucurbita maxima*, *Cucurbita pepo*, *Cucurbita moschata*, *Sechium edule* (Chayote), and *Momordica charantia* (Bitter gourd, red). The homologs peptide in *Nicotiana attenuate* was used as an outgroup. Number at each branch point is the probability to support the tree based on 1000 bootstrapping tests.

Table S1. Information of primers used in this study.

Marker	Assmebly position in draft genome OHB3-1	Chromosome	Position in Dali-11	Position in OHB3-1	Purposes	Forward	Reverse	Enzymes
CAPS1	NW_019104493.1: 986946	Chr4	22447702	25249121	mapping	GTTCCTTATCGGTCGGGTGG	ACTTCAGCGGCGACTACAAA	CAPS (EcoRI)
CAPS2	NW_019104493.1: 862490	Chr4	22323104	25124457	mapping	ACGCCCCGAGGGAAAATATG	TCCCTCTGGTCACTTCGCTA	CAPS (HpaII)
CAPS3	NW_019104493.1: 659446	Chr4	22120019	24921253	mapping	AGGTTTTTCCTTGTTCGGCT	GCTTAGTGGTCATTGGGGCA	CAPS (Eco130I)
CAPS4	NW_019104493.1: 564175	Chr4	22026205	24827295	mapping	ACATGGACCAAAACCAACACT	TGAGACCTCGAATGTCTGACA	CAPS (NdeI)
CAPS5	NW_019104493.1: 527540	Chr4	21989617	24790666	mapping	TGGAGGGATTTGGAGAGATG	TCAAGAAAAATCTGGATGAAGATAAA	CAPS (NdeI)
CAPS6	NW_019104493.1: 478993	Chr4	21940858	24741976	mapping	AAGTTGAGCAAGAAAGACAGACA	GGTTGTTTCCTCCCCATTTA	CAPS (Bsh1236I)
CAPS7	NW_019104493.1: 453676	Chr4	21915520	24716659	mapping	CCGATCCACAGGGAAGTATT	GCAATATCAGTGGATTGATTGAA	CAPS (PvuI)
CAPS8	NW_019104493.1: 446379	Chr4	21908233	24709370	mapping	TGGTTGGATTCACCCTTTGT	GCTTCCTAAATTCAATGCTTGC	CAPS (Hpa II)
SNPM	NW_019104493.1: 436750	Chr4	21898600	24699734	mapping	GACACAGGAATGCAACACAAA	TTGAGGATTGCTTTCCCCCT	sequencing
CAPS9	NW_019104493.1: 343850	Chr4	21805425	24606900	mapping	TCGTGTTGCCCAAGCAATTT	TTCTAAATGATCCCTATGGTGTGAT	CAPS (RsaI)
CAPS10	NW_019104505.1: 63075	Chr4	21376312	24136814	mapping	CCCAGGGGTGGACTCAATTC	CCCGTATTGTAAGGTATATCACGC	CAPS (HpaII)
CAPS11	NW_019104505.1: 845601	Chr4	20592821	23355410	mapping	CCCACCAATCCATCAACCCA	AAGCCACGACACTGCATCTC	CAPS (TaqI)
CAPS12	NW_019104505.1: 1897605	Chr4	19541595	22304742	mapping	TGACATTGTGACCATTGGAAGC	AACGAGGGTGGGTTTCTTGT	CAPS (TaqI)
cEPFL2	NW_019104493.1:464991--465816	Chr4	21926857--21927681	24727974-24728799	cDNA cloning of McEPFL2	TCATTCTTCCAGGCTTCTGCA	AGTGCTAAGTGCCAACCCAA	n/a
gEPFL2	NW_019104493.1:463643--465993	Chr4	21925509--21927858	24726626-24728976	gDNA Cloning of McEPFL2	CTTGCCAAATCATGTGATCCT	GACATGTTTCGGGGTTGTCT	n/a
qEPFL2	NW_019104493.1:465234-465700	Chr4	21927100--21927566	24728217--24728683	qrt-PCR of McEPFL2	ATGCAACATCTCGCTGGGAA	CCAAGTACTTCTTTAGCCCTTTCC	n/a
McCYP2	NW_019104495.1:2013992--2014166	Chr4	MC04: 3266859--3267033	BLBB01000004.1:3265865--3266039	internal reference gene	GGCAAACCCTAAAGTTTTCTTCG	GATGAGCCCTTGTAATGAAGTGG	n/a

Table S2 Statistics of sequencing date

Sample	Sequening type	Sequencing platforms	Raw Reads	Clean Reads	Raw Base(G)	Clean Base(G)	Error Rate(%)	Q20(%)	Q30(%)	GC Content(%)
Z-1-4	DNA requencing	HiSeq-PE250	9047486	9038033	2.71	2.71	0.02	96.85	91.04	36.27
Y1	DNA requencing	HiSeq-PE250	11594584	11582832	3.48	3.48	0.02	97.34	92.15	36.58
DCR bulk	BSA-seq	HiSeq-PE250	30645984	30603029	9.19	9.18	0.02	97.03	91.43	36.45
CR bulk	BSA-seq	HiSeq-PE250	32188807	32153773	9.66	9.65	0.02	97.22	91.84	36.41
Z-1-4	RNA-seq	HiSeq-PE150	50405510	50022448	7.61	7.49	0.02	98.44	94.79	47.34
Y1	RNA-seq	HiSeq-PE150	55629982	55207210	8.40	8.26	0.02	98.48	94.91	47.33

Table S3. List of 100 SNPs with the highest Δ(SNP-index) values between CR and DCR pools.

Scaffold	POS	Ref	CR bulk	DCR bulk	Δ(SNP-index)
NW_019104493.1	18,641	C	0.97	0.16	0.808
NW_019104493.1	19,514	G	1.00	0.17	0.829
NW_019104493.1	74,458	T	1.00	0.12	0.882
NW_019104493.1	82,919	A	1.00	0.16	0.839
NW_019104493.1	83,705	T	1.00	0.06	0.936
NW_019104493.1	109,129	T	1.00	0.19	0.815
NW_019104493.1	129,838	A	1.00	0.17	0.833
NW_019104493.1	137,446	A	1.00	0.15	0.854
NW_019104493.1	161,701	T	1.00	0.12	0.880
NW_019104493.1	223,939	K	1.00	0.17	0.833
NW_019104493.1	243,319	C	1.00	0.19	0.809
NW_019104493.1	343,291	C	1.00	0.13	0.870
NW_019104493.1	442,336	C	1.00	0.18	0.818
NW_019104493.1	446,254	A	1.00	0.20	0.800
NW_019104493.1	446,270	G	1.00	0.20	0.800
NW_019104493.1	446,291	G	1.00	0.20	0.800
NW_019104493.1	446,305	C	1.00	0.19	0.807
NW_019104493.1	475,770	A	1.00	0.18	0.818
NW_019104493.1	476,004	C	1.00	0.20	0.800
NW_019104493.1	478,993	C	1.00	0.18	0.824
NW_019104493.1	490,413	T	1.00	0.17	0.833
NW_019104493.1	553,699	T	1.00	0.21	0.793
NW_019104493.1	564,474	C	1.00	0.20	0.800
NW_019104493.1	572,112	A	1.00	0.20	0.796
NW_019104493.1	659,446	G	1.00	0.19	0.807
NW_019104493.1	659,761	A	1.00	0.21	0.790
NW_019104493.1	668,833	C	1.00	0.21	0.790
NW_019104493.1	869,528	C	1.00	0.21	0.792
NW_019104493.1	880,796	G	1.00	0.19	0.813
NW_019104493.1	1,013,296	G	0.89	0.03	0.853
NW_019104493.1	1,787,329	C	0.94	0.05	0.888
NW_019104505.1	64,561	T	1.00	0.16	0.840
NW_019104505.1	79,744	A	1.00	0.18	0.824
NW_019104505.1	79,745	T	1.00	0.18	0.818
NW_019104505.1	84,073	R	1.00	0.21	0.793
NW_019104505.1	110,151	T	1.00	0.19	0.810
NW_019104505.1	134,641	Y	1.00	0.19	0.808
NW_019104505.1	175,360	Y	1.00	0.17	0.833
NW_019104505.1	197,443	A	1.00	0.18	0.818
NW_019104505.1	308,601	A	0.97	0.12	0.853
NW_019104505.1	402,839	R	1.00	0.19	0.806
NW_019104505.1	406,714	R	1.00	0.18	0.818
NW_019104505.1	472,435	T	1.00	0.14	0.862
NW_019104505.1	472,439	A	1.00	0.11	0.893
NW_019104505.1	472,617	G	0.94	0.06	0.871
NW_019104505.1	539,399	Y	0.98	0.13	0.842
NW_019104505.1	638,658	A	1.00	0.19	0.808
NW_019104505.1	709,442	R	0.97	0.16	0.812
NW_019104505.1	709,476	R	1.00	0.19	0.815
NW_019104505.1	712,616	T	1.00	0.19	0.806
NW_019104505.1	724,273	Y	0.96	0.13	0.828
NW_019104505.1	726,123	R	0.96	0.16	0.798
NW_019104505.1	726,355	R	1.00	0.19	0.808
NW_019104505.1	833,473	R	1.00	0.09	0.913

NW_019104505.1	837,485	C	0.98	0.16	0.821
NW_019104505.1	840,375	C	1.00	0.14	0.857
NW_019104505.1	840,405	A	1.00	0.16	0.840
NW_019104505.1	841,074	A	1.00	0.20	0.800
NW_019104505.1	841,896	C	1.00	0.13	0.875
NW_019104505.1	844,627	G	0.98	0.12	0.854
NW_019104505.1	845,565	C	1.00	0.16	0.839
NW_019104505.1	899,585	C	0.95	0.16	0.793
NW_019104505.1	1,016,368	C	1.00	0.18	0.821
NW_019104505.1	1,016,427	A	0.97	0.13	0.840
NW_019104505.1	1,016,428	A	0.97	0.14	0.836
NW_019104505.1	1,122,412	R	1.00	0.20	0.805
NW_019104505.1	1,122,903	R	0.95	0.14	0.817
NW_019104505.1	1,177,262	G	1.00	0.21	0.795
NW_019104505.1	1,177,276	Y	1.00	0.14	0.857
NW_019104505.1	1,243,581	T	0.96	0.11	0.859
NW_019104505.1	1,244,476	A	1.00	0.19	0.813
NW_019104505.1	1,248,669	A	0.90	0.10	0.810
NW_019104505.1	1,249,000	R	1.00	0.21	0.792
NW_019104505.1	1,257,737	G	1.00	0.18	0.818
NW_019104505.1	1,349,066	G	1.00	0.14	0.857
NW_019104505.1	1,469,413	T	0.96	0.14	0.818
NW_019104505.1	1,469,445	C	0.95	0.13	0.818
NW_019104505.1	1,474,465	A	1.00	0.16	0.840
NW_019104505.1	1,525,899	T	1.00	0.16	0.839
NW_019104505.1	1,612,282	T	1.00	0.17	0.833
NW_019104505.1	1,612,289	A	1.00	0.18	0.821
NW_019104505.1	1,612,373	T	1.00	0.15	0.852
NW_019104505.1	1,612,510	T	0.97	0.15	0.826
NW_019104505.1	1,682,407	G	0.97	0.15	0.818
NW_019104505.1	1,990,602	G	1.00	0.16	0.840
NW_019104505.1	2,191,543	T	1.00	0.18	0.818
NW_019104528.1	836,643	W	0.97	0.14	0.833
NW_019104528.1	1,194,131	T	1.00	0.17	0.826
NW_019104528.1	1,200,461	T	0.95	0.15	0.801
NW_019104528.1	1,200,463	G	0.98	0.18	0.800
NW_019104528.1	1,200,595	C	0.97	0.18	0.793
NW_019104528.1	1,284,356	Y	0.95	0.10	0.854
NW_019104542.1	566,476	C	0.97	0.12	0.849
NW_019104542.1	629,834	T	0.79	0.00	0.794
NW_019104575.1	232,144	G	0.95	0.10	0.855
NW_019104613.1	89,488	M	0.93	0.13	0.796
NW_019104613.1	116,982	K	0.84	0.04	0.807
NW_019104632.1	330,499	C	0.90	0.10	0.805
NW_019104719.1	246,839	A	0.83	0.03	0.795
NW_019104804.1	29,085	G	0.93	0.12	0.805

Table S4. Predicted genes in the cr candidate gene region between molecular markers SNPM and CAP5

Gene ID	Scaffold	start	end	strand	Predicted functions
gene3920	NW_019104493.1	419,923	423,067	-	G-type lectin S-receptor-like serine/threonine-protein kinase
gene3921	NW_019104493.1	425,331	431,436	-	putative CCA tRNA nucleotidyltransferase 2
gene3922	NW_019104493.1	432,081	435,092	-	alpha-(1,4)-fucosyltransferase
gene3923	NW_019104493.1	435,777	442,591	-	uncharacterized
gene3924	NW_019104493.1	447,908	452,539	+	receptor protein-tyrosine kinase CEPR2
gene3925	NW_019104493.1	453,772	458,985	-	adenylate kinase, chloroplastic
gene3926	NW_019104493.1	462,863	463,577	+	type 1 phosphatases regulator ypi1
gene3927	**NW_019104493.1**	**463,692**	**465,933**	**-**	**protein EPIDERMAL PATTERNING FACTOR 2-like**
gene3928	NW_019104493.1	468,929	474,683	+	uncharacterized
gene3929	NW_019104493.1	474,475	479,021	-	calcium and calcium/calmodulin-dependent serine/threonine-protein kinase-like
gene3930	NW_019104493.1	481,317	485,012	-	ETHYLENE INSENSITIVE 3-like 1 protein
gene3931	NW_019104493.1	494,857	499,737	-	type I inositol polyphosphate 5-phosphatase 10
gene3932	NW_019104493.1	503,686	508,511	-	protein SAD1/UNC-84 domain protein 1
gene3933	NW_019104493.1	509,782	515,540	-	ABSCISIC ACID-INSENSITIVE 5-like protein 2
gene3934	NW_019104493.1	517,172	520,392	-	uncharacterized
gene3935	NW_019104493.1	521,756	526,939	-	transcription factor MYB33-like

Table S5. Annotation of SNP and INDEL by snpEFF

Scaffold	Pos	Ref	Alt	DCR bulk	CR bulk	Y1	Z-1-4	Annotation	Putative_impact	Gene(in or nea	Rank/total	HGVS.c	HGVS.p	cDNA.pos/cDNA.len	CDS.pos/CDS.len	AA.pos/AA.length
NW_019104493.1	435,199	T	A	T/A	T/T	T/T	A/A	upstream_gene_variant	MODIFIER	gene3921		c.-3904A>T				
NW_019104493.1	435,628	A	G	A/G	A/A	A/A	G/G	upstream_gene_variant	MODIFIER	gene3921		c.-4333T>C				
NW_019104493.1	436,248	G	A	G/A	G/G	G/G	A/A	synonymous_variant	LOW	gene3923	10/10	c.2460C>T	p.Asp820Asp	2656/3127	2460/2757	820/918
NW_019104493.1	436,389	C	T	C/T	C/C	C/C	T/T	synonymous_variant	LOW	gene3923	10/10	c.2319G>A	p.Leu773Leu	2515/3127	2319/2757	773/918
NW_019104493.1	436,750	C	T	C/T	T/T	T/T	C/C	upstream_gene_variant	MODIFIER	gene3923		c.-1787G>A				
NW_019104493.1	438,029	G	A	G/A	A/A	A/A	G/G	upstream_gene_variant	MODIFIER	gene3922		c.-3066C>T				
NW_019104493.1	441,469	A	G	A/G	A/A	A/A	G/G	intron_variant	MODIFIER	gene3923	2/9	c.317+269T>C				
NW_019104493.1	442,336	C	T	C/T	C/C	C/C	T/T	synonymous_variant	LOW	gene3923	1/10	c.60G>A	p.Pro20Pro	256/3127	60/2757	20/918
NW_019104493.1	442,804	G	A	G/A	G/G	G/G	A/A	upstream_gene_variant	MODIFIER	gene3923		c.-409C>T				
NW_019104493.1	443,588	C	G	C/G	G/G	G/G	C/C	upstream_gene_variant	MODIFIER	gene3923		c.-1193G>C				
NW_019104493.1	443,985	TA	T	TA/T	TA/T	T/T	TA/TA	upstream_gene_variant	MODIFIER	gene3923		c.-1591delT				
NW_019104493.1	444,903	TA	T	TA/T	T/T	T/T	TA/TA	upstream_gene_variant	MODIFIER	gene3923		c.-2517delT				
NW_019104493.1	445,028	C	A	C/A	A/A	A/A	C/C	upstream_gene_variant	MODIFIER	gene3923		c.-2633G>T				
NW_019104493.1	445,379	T	TTC	T/TTC	TTC/TTC	TTC/TTC	T/T	upstream_gene_variant	MODIFIER	gene3923		c.-3092delT				
NW_019104493.1	445,479	GA	G	GA/G	G/G	G/G	GA/GA	upstream_gene_variant	MODIFIER	gene3923		c.-3092delT				
NW_019104493.1	445,532	G	A	G/A	A/A	A/A	G/G	upstream_gene_variant	MODIFIER	gene3923		c.-3137C>T				
NW_019104493.1	445,754	C	CT	C/CT	C/C	C/C	CT/CT	upstream_gene_variant	MODIFIER	gene3923		c.-3368_-3367insA				
NW_019104493.1	445,888	G	A	G/A	A/A	A/A	G/G	upstream_gene_variant	MODIFIER	gene3923		c.-3493C>T				
NW_019104493.1	445,961	AT	A	AT/A	AT/AT	AT/AT	A/A	upstream_gene_variant	MODIFIER	gene3923		c.-3572delA				
NW_019104493.1	445,997	G	T	G/T	T/T	T/T	G/G	upstream_gene_variant	MODIFIER	gene3923		c.-3602C>A				
NW_019104493.1	446,033	AC	A	AC|A	AC/AC	AC/AC	A|A	upstream_gene_variant	MODIFIER	gene3923		c.-3639delG				
NW_019104493.1	446,249	AAC	A	AAC|A	A|A	A|A	AAC/AAC	upstream_gene_variant	MODIFIER	gene3923		c.-3856_-3855delGT				
NW_019104493.1	446,496	GTATT	G	GTATT/G	G/G	G/G	GTATT/GTATT	upstream_gene_variant	MODIFIER	gene3923		c.-4113_-4110delAATA				
NW_019104493.1	447,905	A	ACT	A/ACT	ACT/ACT	ACT/ACT	A/A	5_prime_UTR_variant	MODIFIER	gene3924	1/3	c.-767_-766dupTC				
NW_019104493.1	447,981	T	A	T|A	A|A	A|A	T/T	5_prime_UTR_variant	MODIFIER	gene3924	1/3	c.-701T>A				
NW_019104493.1	448,003	TC	T	TC|T	T|T	T|T	TC/TC	5_prime_UTR_variant	MODIFIER	gene3924	1/3	c.-677delC				
NW_019104493.1	448,090	C	T	C|T	T|T	T|T	C/C	5_prime_UTR_premature_start_co	LOW	gene3924	1/3	c.-592C>T				
NW_019104493.1	448,092	A	T	A|T	T|T	T|T	A/A	5_prime_UTR_variant	MODIFIER	gene3924	1/3	c.-590A>T				
NW_019104493.1	448,295	G	A	G/A	A/A	A/A	G/G	5_prime_UTR_variant	MODIFIER	gene3924	1/3	c.-387G>A				
NW_019104493.1	449,998	T	C	T/C	C/C	C/C	T/T	synonymous_variant	LOW	gene3924	2/3	c.876T>C	p.Phe292Phe	1650/3934	876/2934	292/977
NW_019104493.1	450,103	C	T	C/T	T/T	T/T	C/C	synonymous_variant	LOW	gene3924	2/3	c.981C>T	p.Phe327Phe	1755/3934	981/2934	327/977
NW_019104493.1	452,824	G	T	G/T	T/T	T/T	G/G	upstream_gene_variant	MODIFIER	gene3925		c.-947G>T				
NW_019104493.1	452,994	C	G	C/G	G/G	G/G	C/C	upstream_gene_variant	MODIFIER	gene3925		c.-777C>G				
NW_019104493.1	453,132	C	T	C/T	T/T	T/T	C/C	upstream_gene_variant	MODIFIER	gene3925		c.-639C>T				
NW_019104493.1	453,319	G	T	G/T	T/T	T/T	G/G	upstream_gene_variant	MODIFIER	gene3925		c.-452G>T				
NW_019104493.1	453,411	C	G	C/G	G/G	G/G	C/C	upstream_gene_variant	MODIFIER	gene3925		c.-360C>G				
NW_019104493.1	453,514	A	T	A/T	T/T	T/T	A/A	upstream_gene_variant	MODIFIER	gene3925		c.-257A>T				
NW_019104493.1	453,577	T	C	T/C	C/C	C/C	T/T	upstream_gene_variant	MODIFIER	gene3925		c.-194T>C				
NW_019104493.1	453,696	AATGC	A	AATGC/A	A/A	A/A	AATGC/AATGC	upstream_gene_variant	MODIFIER	gene3925		c.-72_-69delGCAT				
NW_019104493.1	454,490	G	C	G/C	C/C	C/C	G/G	downstream_gene_variant	MODIFIER	gene3924		c.*1951G>C				
NW_019104493.1	455,339	G	A	G/A	A/A	A/A	G/G	downstream_gene_variant	MODIFIER	gene3924		c.*2800G>A				
NW_019104493.1	455,401	A	G	A/G	G/G	G/G	A/A	synonymous_variant	LOW	gene3925	5/7	c.696T>C	p.Thr232Thr	915/1446	696/885	232/294
NW_019104493.1	455,778	T	C	T/C	C/C	C/C	T/T	downstream_gene_variant	MODIFIER	gene3924		c.*3239T>C				
NW_019104493.1	456,401	AG	A	AG/A	A/A	A/A	AG/AG	downstream_gene_variant	MODIFIER	gene3924		c.*3864delG				
NW_019104493.1	456,548	C	G	C/G	G/G	G/G	C/C	downstream_gene_variant	MODIFIER	gene3924		c.*4009C>G				
NW_019104493.1	456,823	GA	G	GA/G	G/G	G/G	GA/GA	downstream_gene_variant	MODIFIER	gene3924		c.*4294delA				
NW_019104493.1	457,115	A	G	A/G	G/G	G/G	A/A	downstream_gene_variant	MODIFIER	gene3924		c.*4576A>G				
NW_019104493.1	457,345	T	A	T/A	A/A	A/A	T/T	downstream_gene_variant	MODIFIER	gene3924		c.*4806T>A				
NW_019104493.1	458,779	TCCGCCG	T	TCCGCCGATCT	TCCGCCGATCT	TCCGCCGATCT	T/T	5_prime_UTR_variant	MODIFIER	gene3925	1/7	c.-35_-20delCGGCGGCGGAGGAGAT				
NW_019104493.1	460,561	T	TG	T/TG	TG/TG	TG/TG	T/T	upstream_gene_variant	MODIFIER	gene3925		c.-1796_-1795insC				
NW_019104493.1	460,673	C	CCTC	C/CCTCCTTATA	CCTCCTTATAT	CCTCCTTATAT	C/C	upstream_gene_variant	MODIFIER	gene3925		c.-1921_-1920insCCCATATAAGGAG				
NW_019104493.1	460,959	G	T	G/T	T/T	T/T	G/G	upstream_gene_variant	MODIFIER	gene3925		c.-2193C>A				
NW_019104493.1	461,613	A	T	A/T	T/T	T/T	A/A	upstream_gene_variant	MODIFIER	gene3925		c.-2847T>A				
NW_019104493.1	461,848	T	C	T/C	C/C	C/C	T/T	upstream_gene_variant	MODIFIER	gene3925		c.-3082A>G				
NW_019104493.1	461,935	A	G	A/G	G/G	G/G	A/A	upstream_gene_variant	MODIFIER	gene3925		c.-3169T>C				
NW_019104493.1	462,118	C	T	C/T	T/T	T/T	C/C	upstream_gene_variant	MODIFIER	gene3925		c.-3352G>A				
NW_019104493.1	462,676	A	G	A/G	G/G	G/G	A/A	upstream_gene_variant	MODIFIER	gene3925		c.-3910T>C				
NW_019104493.1	462,727	G	A	G/A	A/A	A/A	G/G	upstream_gene_variant	MODIFIER	gene3925		c.-3961C>T				
NW_019104493.1	462,816	A	G	A/G	G/G	G/G	A/A	upstream_gene_variant	MODIFIER	gene3925		c.-4050T>C				
**NW_019104493.1**	**465,777**	**CT**	**C**	**CT/C**	**C/C**	**C/C**	**CT/CT**	**frameshift_variant**	**HIGH**	**gene3927**	**1/3**	**c.4delA**	**p.Lys2fs**	**155/1879**	**4/357**	**2/118**
NW_019104493.1	474,455	C	A	C/A	A/A	A/A	C/C	upstream_gene_variant	MODIFIER	gene3929		c.-19C>A				
NW_019104493.1	475,770	A	C	A/C	C/C	C/C	A/A	downstream_gene_variant	MODIFIER	gene3928		c.*1876A>C				
NW_019104493.1	476,004	C	T	C/T	T/T	T/T	C/C	downstream_gene_variant	MODIFIER	gene3928		c.*2110C>T				
NW_019104493.1	476,209	A	T	A/T	T/T	T/T	A/A	synonymous_variant	LOW	gene3929	5/7	c.1257T>A	p.Ile419Ile	1638/2104	1257/1554	419/517
NW_019104493.1	478,596	T	C	T/C	C/C	C/C	T/T	synonymous_variant	LOW	gene3929	1/7	c.45A>G	p.Ser15Ser	426/2104	45/1554	15/517
NW_019104493.1	478,706	A	T	A/T	T/T	T/T	A/A	5_prime_UTR_variant	MODIFIER	gene3929	1/7	c.-66T>A				
NW_019104493.1	478,742	G	A	G/A	A/A	A/A	G/G	5_prime_UTR_premature_start_co	LOW	gene3929	1/7	c.-102C>T				
NW_019104493.1	478,993	C	T	C/T	T/T	T/T	C/C	5_prime_UTR_variant	MODIFIER	gene3929	1/7	c.-353G>A				

NW_019104493.1	479,206	G	A	G/A	A/A	A/A	G/G	upstream_gene_variant	MODIFIER	gene3929		c.-566C>T				
NW_019104493.1	479,700	A	ATG	A/ATG	ATG/ATG	ATG/ATG	A/A	upstream_gene_variant	MODIFIER	gene3929		c.-1062_-1061insAC				
NW_019104493.1	480,635	GT	G	GT/G	G/G	G/G	GT/GT	upstream_gene_variant	MODIFIER	gene3929		c.-2003delA				
NW_019104493.1	483,348	A	G	A/G	G/G	G/G	A/A	synonymous_variant	LOW	gene3930	3/3	c.132T>C	p.Ser44Ser	894/2925	132/1848	44/615
NW_019104493.1	484,440	T	A	T/A	A/A	A/A	T/T	5_prime_UTR_premature_start_co	LOW	gene3930	1/3	c.-394A>T				
NW_019104493.1	485,780	T	C	T/C	C/C	C/C	T/T	upstream_gene_variant	MODIFIER	gene3930		c.-1734A>G				
NW_019104493.1	487,763	GT	G	GT/G	G/G	G/G	GT/GT	upstream_gene_variant	MODIFIER	gene3930		c.-3727delA				
NW_019104493.1	488,151	T	TA	T/TA	TA/TA	TA/TA	T/T	upstream_gene_variant	MODIFIER	gene3930		c.-4115_-4114insT				
NW_019104493.1	490,413	T	C	T/C	C/C	C/C	T/T	upstream_gene_variant	MODIFIER	gene3931		c.-4443T>C				
NW_019104493.1	492,453	C	A	C/A	A/A	A/A	C/C	upstream_gene_variant	MODIFIER	gene3931		c.-2403C>A				
NW_019104493.1	493,258	T	C	T/C	C/C	C/C	T/T	upstream_gene_variant	MODIFIER	gene3931		c.-1598T>C				
NW_019104493.1	504,849	G	GA	G/GA	G/GA	GA/GA	G/G	upstream_gene_variant	MODIFIER	gene3933		c.-4932_-4931insA				
NW_019104493.1	509,229	A	AT	A/AT	AT/AT	AT/AT	A/A	upstream_gene_variant	MODIFIER	gene3932		c.-980_-979insA				
NW_019104493.1	509,536	A	G	A/G	G/G	G/G	A/A	upstream_gene_variant	MODIFIER	gene3932		c.-1278T>C				
NW_019104493.1	527,292	T	A	T/A	A/A	A/A	T/T	upstream_gene_variant	MODIFIER	gene3935		c.-578A>T				

Table S6. Statistics of variants annotated with different mutant types

Annotation	Number	Percentages
upstream_gene_variant	51	61.45%
downstream_gene_variant	10	12.05%
5_prime_UTR_variant	8	9.64%
5_prime_UTR_premature_start_codon_gain_variant	3	3.61%
intron_variant	1	1.20%
synonymous_variant	9	10.84%
frameshift_variant	1	1.20%
total	83	100.00%

Table S7. Expression of genes in the cr candidate gene region in Z-1-4 and Y1 by RNA-seq (Y1 vs Z-1-4)

Gene ID	FPKM_Z-1-4	FPKM_Y1	log2(FC)	P-value	Padjust	Significance	Expression
gene3920	4.581	6.848	0.75	0.000	0.000	no	up
gene3921	13.249	14.062	0.29	0.112	0.193	no	up
gene3922	32.593	32.991	0.19	0.508	0.619	no	up
gene3923	23.13	44.02	**1.10**	**0.000**	**0.000**	**yes**	up
gene3924	22.36	23.70	0.24	0.041	0.085	no	up
gene3925	47.14	70.97	0.76	0.000	0.000	no	up
gene3926	83.03	76.62	-0.05	0.243	0.354	no	down
gene3927	**1.12**	**0.34**	**-1.54**	**0.000**	**0.001**	**yes**	**down**
gene3928	10.57	7.67	-0.29	0.000	0.000	no	down
gene3929	10.11	3.31	**-1.44**	**0.000**	**0.000**	**yes**	down
gene3930	70.26	73.32	0.23	0.011	0.029	no	up
gene3931	0.08	0.27	1.59	0.109	0.188	no	up
gene3932	46.04	55.53	0.44	0.000	0.000	no	up
gene3933	40.92	39.62	-0.13	0.794	0.856	no	down
gene3934	17.28	16.34	-0.09	0.640	0.733	no	down
gene3935	5.19	4.67	-0.01	0.286	0.401	no	down

Table S8. Protein sequences of McEPFL2 homologs from 10 plant species.

>XP_022134337.1 Momordica charantia
MKKCSLVAHLSFIFVVFFIILMIGRSLDATSRWEHMSFNAEDIHRTVDSSMHQKERAKEVLGMELYPTGSSLPDCSHACGPCFP
CKRVMVSFKCSVAESCPTVYRCMCKGKYYHVPSN

>XP_019228875.1 Nicotiana attenuata
MVTTTHSFGFLILSIVTLVLMLVASTQSLRPYHFTYHGKGTNMDKQSHVHIKEERKGELGMELYPTGSSLPDCSHACGPCFPCK
RVMVSFECSIAESCPIVYRCMCRGKYYHVPSN

>XP_038885051.1 Benincasa hispida
MKSFPLVAHTSILFGIFFIILMNGRSLDATTRWDHMSFNAEDTHRAVDNSRHEQEKTEEVLGMELYPTGSSLPDCSHACGPCF
PCKRVMVSFKCSVAESCPTVYKCMCKGKYYHVPSN

>XP_004140931.1 Cucumis sativus
MKRLPLVAHISLLFVIFFIILMIGSSLDATRWDHMSFNAEDTHRTINHSRHQQEKTKEVLGMELYPTGSSLPDCSHACGPCFPC
KRVMVSFKCSVAESCPTVYRCMCKGKYYRVPSN

>XP_023550443.1 Cucurbita pepo subsp. pepo
MWKFPLVAQTSLLFWIFFIILMIGRRLDASTRWDHMSFNVEDTHRPVGSSTHQEEKTEEALGMELYPTGSNLPDCSHACGPC
FPCKRVMVSFKCSAAESCPTVYRCMCKGKYYHVPSN

>XP_022992672.1 Cucurbita maxima
MKKFPLVAQTLLFWIFFIILMIGRRLDASTRWDHMRFNAEDTHRPVGSSTHQEEKTEEALGMELYPTGSSLPDCSHACGPCFP
CKRVMVSFKCSVAESCPTVYRCMCKGKYYHVPSN

>XP_022939743.1 Cucurbita moschata
MKKFPLVAQTSLLFWIFFIILMIGRRLDASTHWDHMSFNAEDTHRPVGSSTHHEEKTEALGMELYPTGSSLPDCSHACGPCFP
CKRVMVSFKCSAAESCPTVYRCMCKGKYYHVPSN

>Cla97C08G151460.2 Citrullus lanatus
MKKFQLVPHTSLLFGIFFIILMIGRSLDATTRWDHMSFNAEDTHRTVGNSRHQQEKTKEVLGMELYPTGSSLPDCSHACGPCF
PCKRVMVSFKCSVAESCPTVYRCMCKGKYYHVPSN

>MELO3C019934.2.1 Cucumis melon
VTNISNKLCSLFLPPCFYHFLYPFGSCFYQTKMKRFPLVAHISLLFGIFFIILMIGRSLDAARWDHMSFNAEDTHRTVNNSRNQQ
EKTKEVLGMELYPTGSSLPDCSHACGPCFPCKRVMVSFKCSVAESCPTVYRCMCKGKYYHVPSN

>Sed0027653.1 Sechium edule
MKKFSLLAHTSLLFGIFFIILMIGRSLDATTRWDHLSSNAEHTHKTVRSSRPQKEKAKEVLGMELYPTGSSLPDCSHACGPCFPC
KRVMVSFKCSVAESCPTVYRCMGNGKYYHVPSN
